# Sleep During NICU Care Is Associated With Proportional Cerebellar Size at Term-Equivalent Age in Extremely Preterm Infants

**DOI:** 10.1007/s12311-026-01988-2

**Published:** 2026-04-10

**Authors:** J. M. Brouwer, A. M. Bakker, E. de Groot, M.L. Tataranno, M. Benders, J. Dudink

**Affiliations:** 1 Faculty of Science, University of Amsterdam, Science Park 904, Amsterdam, 1098 XH The Netherlands; 2 Department of Neonatology, Wilhelmina Children’s Hospital, University Medical Center Utrecht, Lundlaan 6, Utrecht, 3584 EA The Netherlands; 3 Faculty of Medical Sciences, University of Groningen, Antonius Deusinglaan 1, Groningen, 9713 AV The Netherlands

**Keywords:** Sleep, Premature, Cerebellum development, Neurodevelopmental disorders

## Abstract

**Background:**

Infants born extremely preterm (< 28 weeks’ gestation) are born during a phase of rapid cerebellar growth, when disturbances in brain maturation are common and reduced cerebellar size has been linked to unfavorable neurodevelopment. During late gestation, sleep is the dominant brain state and provides structured endogenous activity supporting circuit formation. In the neonatal intensive care unit (NICU), sleep may become fragmented at a developmental stage when the cerebellum is particularly vulnerable. We hypothesized that sleep quantity during late NICU care is associated with proportional cerebellar size at term-equivalent age (TEA).

**Methods:**

In a retrospective cohort of extremely preterm infants, continuous cardiorespiratory signals recorded at approximately 29–32 weeks postmenstrual age were analyzed using a validated algorithm to quantify sleep–wake states. All infants underwent 3 T MRI at TEA. The primary outcome was cerebellum-to-total brain volume ratio. Associations were tested using multivariable linear regression adjusted for age at MRI, birthweight, duration of mechanical ventilation, and sex.

**Results:**

Seventy-one infants contributed multi-day sleep recordings and TEA MRI. Higher total sleep percentage during late NICU monitoring was associated with a larger cerebellum-to-total brain volume ratio at TEA after adjustment for clinical covariates (p = 0.011). Sleep-state composition was not associated with absolute cerebellar volume, which was primarily related to clinical factors.

**Conclusions:**

Greater total sleep during late NICU care is associated with proportionally larger cerebellar size at term-equivalent age. Further studies are needed to determine whether sleep represents a modifiable factor influencing cerebellar development or a marker of overall physiological stability.

## Introduction

Unlike full-term infants, children born extremely preterm (< 28 weeks’ gestation) complete much of late second- and third-trimester brain development in the neonatal intensive care unit rather than in the intrauterine environment. This period coincides with rapid brain growth and heightened plasticity, during which neural circuits are actively formed and refined, rendering the preterm brain particularly susceptible to altered sensory input, physiological stress, and illness. By term-equivalent age (TEA), these early extrauterine influences are already detectable on MRI. Cerebellar size at TEA reflects biologically relevant variation and has been consistently associated with later cognitive and behavioral outcomes, with extremely preterm birth linked to smaller cerebellar volumes and less favorable long-term development [1–3].

Neonatal MRI studies further demonstrate that brain measures obtained at TEA, including cerebellar metrics, are associated with later neurodevelopmental performance. Longitudinal follow-up of children born preterm shows that reduced cerebellar volume, altered growth trajectories and disrupted regional maturation during this period are linked to persistent cognitive and motor difficulties across childhood [4, 5]. Together, these findings support the use of TEA cerebellar volume as a practical surrogate marker for later functioning and underline the particular vulnerability of the cerebellum in extremely preterm infants during this developmental window [6, 7].

In typical late gestation, the cerebellum shows very rapid growth, outpacing the rest of the brain, driven largely by granule-cell proliferation and migration in the external granular layer [8–10]. When this tightly timed program is shifted from the womb to the NICU, disruption of growth conditions may help explain why EP infants often have smaller cerebellar volumes even in the absence of overt lesions (such as large hemorrhages). Functional topography studies link anterior lobules mainly to sensorimotor control and posterior regions to higher cognitive functions, mirroring the motor and executive problems frequently observed after preterm birth [11–13].

Across the same developmental window, sleep is the dominant form of brain activity. Active and quiet sleep alternate in long bouts and together occupy most of the 24-hour cycle. Current models view sleep not as a passive state but as a main driver of endogenous brain activity that supports early circuit formation. Active sleep, with its bursts of myoclonic twitches, provides structured, internally generated sensorimotor input that can shape cerebellar and thalamocortical networks, whereas quiet sleep is thought to support synaptic renormalization as overall experience accumulates [14–17]. Animal and human infant work has shown twitch-linked activation of cerebellar cortex and connected sensorimotor pathways, consistent with activity-dependent refinement of these circuits [18–21].

In early infancy, myoclonic twitches occurring throughout active sleep function as discrete sensorimotor events that drive cerebellar calibration and the refinement of the developing body schema. Work from Blumberg and colleagues [15] indicates that twitch-related activity can originate in the red nucleus and is accompanied by corollary discharge signals (internal copies of motor commands) that converge with reafferent sensory input across developing sensorimotor pathways, including cerebellar targets. In this framework, corollary discharge offers a predictive signal, while peripheral feedback provides a teaching signal, together supporting the alignment of cerebellar internal models with the infant’s biomechanics. Chronic fragmentation or suppression of active sleep in the NICU may therefore deprive the cerebellum of this dense, structured sensorimotor “training data,” with possible consequences for circuit tuning and later function [22–24].

The NICU environment differs profoundly from the womb. Light, noise, procedures, mechanical ventilation, and illness can fragment and shorten sleep, altering the very patterns of brain activity thought to guide activity-dependent growth [25]. Observational studies in preterm infants have linked early sleep organization to emerging neurodevelopment, supporting the idea that protecting sleep may be beneficial for brain maturation [26, 27]. Yet direct evidence connecting quantified NICU sleep to cerebellar size at TEA remains limited.

Against this background, we wanted to examine whether sleep during the late second to early third trimester equivalent period (29–31 weeks postmenstrual age (PMA)) is associated with cerebellar development at term-equivalent age in extremely preterm infants. Using a validated algorithm applied to routine bedside cardiorespiratory monitoring, we quantified total sleep time and behavioral state composition (active sleep, quiet sleep, and wake) over multiple days between 29 and 32 weeks postmenstrual age. These sleep measures were related to cerebellar outcomes on MRI at term-equivalent age, with the cerebellum-to-total brain volume ratio as the primary outcome and absolute cerebellar volume as a secondary measure, adjusting for postmenstrual age at MRI, birthweight, duration of ventilation, and sex. We hypothesized that greater total sleep during the late second to early third trimester equivalent period (29–31 weeks PMA) would be associated with a larger proportional cerebellar size at term-equivalent age.

## Patients and Methods

We performed a retrospective cohort study in a level-III NICU (Wilhelmina Children’s Hospital, UMC Utrecht). Physiological signals were captured during routine care in late NICU weeks, and brain MRI was obtained at term-equivalent age. The primary exposure was infant-level sleep quantity derived from an automated vital-signs–based classifier (Sleep Well Baby, SWB) [28].

### Participants

Infants were eligible if born extremely preterm (gestational age < 28 weeks), had continuous cardiorespiratory monitoring between 29 + 0 and 31 + 6 weeks PMA, and underwent TEA MRI (≈ 38–42 weeks PMA) of sufficient quality. Exclusions: major congenital malformations; overt brain injury (e.g., grade III–IV intraventricular hemorrhages (IVH), cystic periventricular leukomalacia (cPVL) and/or infarction); morphine administration or invasive ventilation during sleep monitoring; and > 5 h missing cardiorespiratory monitoring within the observation window. Ethics approval was obtained (MREC UMC Utrecht 21-066-C); data were pseudonymized.

### Physiological monitoring and data handling

Heart rate (HR), respiratory rate (RR), and oxygen saturation (SpO₂) were recorded from patient monitors (Philips IntelliVue MP70, Philips Medizin Systeme Böblingen GmbH, Böblingen, Germany) and archived in BedBase (an in-house developed software system for patient data collection). Vital signs were sampled at 0.4 Hz. For each infant, a consecutive 24-h reference segment within ± 3.5 days of the observation period was required to enable per-infant normalization; infants without such data were excluded from SWB analyses. Epochs with uncertain behavioral labels in the SWB classification were removed.

### Sleep-state computation (SWB workflow)

We implemented the published SWB pipeline: per-infant normalization of HR/RR using a 24-h reference (PMA 28–34 weeks), removal of missing samples, and exclusion of epochs with > 50% feature-window missingness [28]. Minute epochs drew 108 features from HR, RR, and SpO₂ using 60–480 s windows, which a random-forest classifier (with isotonic probability calibration) used to estimate wake (W), active sleep (AS), and quiet sleep (QS). Calibrated probabilities were summed across valid minutes to derive infant-level percent time in state; the primary exposure was total sleep% (AS + QS), with AS% and QS% as secondary.

MRI acquisition

### MRI acquisition

All infants underwent MRI at term-equivalent age on a 3.0 T scanner (Achieva, Philips Medical Systems, Best, The Netherlands) using a SENSE head coil. Sequences included a high-resolution T2-weighted sequence (turbo spin-echo) using parameters: TR ≈ 4851 ms, TE ≈ 150 ms, in-plane 0.35 × 0.35 mm², slice thickness 1.2–2.0 mm. Infants were scanned during natural sleep or with oral chloral hydrate (50–60 mg/kg) if required; immobilization and double hearing protection was used. A neonatologist/PA monitored infants throughout the scanning. Scans were quality-checked by an experienced neuro-neonatologist.

### Image processing and outcomes

**Fig. 1:**
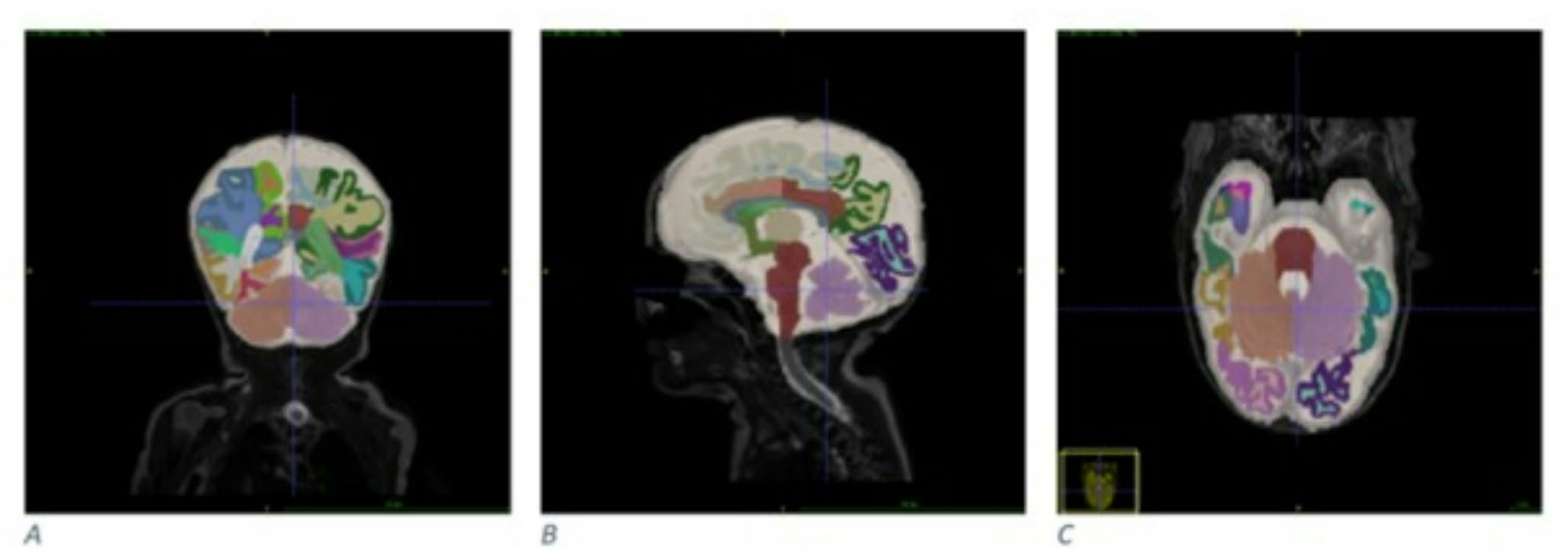
Example of a segmented neonatal brain using the dHCP pipeline on a T2-weighted MRI. (**A**) coronal view, (**B**) sagittal view, and (**C**) axial view. The segmentation includes brain tissue and ventricular spaces, used to extract cerebellar volume and total cranial volume

Structural T2-weighted images were processed with the neonatal structural pipeline of the developing Human Connectome Project (dHCP; [29]) (Fig. 1), including bias correction, brain extraction, and tissue segmentation. The primary outcome was proportional cerebellar size, expressed as the cerebellum-to-brain. The secondary outcome was absolute cerebellar volume.

### Statistical Analysis

All analyses were conducted using IBM SPSS Statistics (version 30.0.0.0). Descriptive statistics were calculated for sleep architecture parameters, cerebellar volume, and relevant clinical characteristics. To identify predictors of relative cerebellar volume at term-equivalent age, a set of theoretically and empirically relevant covariates was evaluated: PMA at MRI scan, birthweight (BW), duration of ventilation (DOV), sex, gestational age at birth (GA), PMA at sleep assessment (PMA_start_sleepdate), and socioeconomic status (SES). Duration of invasive ventilation was included a priori as an integrated marker of illness severity, given its established association with brain growth at term-equivalent age in extremely preterm infants.

Covariate selection was guided by theoretical relevance, bivariate correlations, and multicollinearity diagnostics. GA at birth was excluded due to high collinearity with birthweight (*r* = 0.575, *p* < 0.001), with BW retained for its stronger predictive value. PMA at sleep assessment and SES were excluded due to weak and non-significant associations with cerebellar volume (*r* = –0.212, *p* = 0.076 and *r* = –0.144, *p* = 0.231, respectively). Sensitivity analyses confirmed that exclusion of these variables did not affect model outcomes.

The final regression model included PMA_MRI_days, BW, DOV, and sex. Hierarchical linear regression was applied, and model assumptions (normality, linearity, and multicollinearity) were verified. Standardized regression coefficients (β) and unstandardized B-values with 95% confidence intervals (CI) were reported. Statistical significance was defined as *p* < 0.05, and all p-values were two-sided.

## Results

### Cohort

Seventy-one extremely preterm infants were included (60.6% female). Mean (SD) gestational age at birth was 26.7 (0.8) weeks and birthweight 906 (173) g. Sleep assessment began at 30.0 (0.6) weeks PMA; MRI was obtained at TEA, 41.0 (0.9) weeks PMA. Socio-economic score averaged 0.11 (0.20). Days of mechanical ventilation varied (10.1 [13.6]). Full characteristics are provided in Table 1.

**Table 1:** Patient characteristics of study population. Data are shown in n (%), mean (SD; range), mean (SD), or median (Q1, Q3). Ga, gestational age; PMA, postmenstrual age; TEA, term equivalent age; MRI, magnetic resonance imaging; SES, socio-economic status; DOV, Days of ventilation. There were no cases of NEC, PDA or gram negative sepsis

Patient characteristics	*N* = 71
Female sex, n (%)	43 (60.6%)
GA at birth, weeks	26.7 (0,8; 24.7–27.9)
Birth weight, grams	906 (173)
Apgar scores - At 1st minute - At 5th minute - At 10th minute	5 (2,4; 1–10) 7 (1,5; 3–10) 8 (0,7; 7–10)
PMA at start of sleep assessment, weeks	30,0 (0,6; 29,0–31,1)
PMA at TEA MRI scan, weeks	41,0 (0,9; 39,6–44,1)
SES	0,11 (0,2)
DOV	10,1 (13,6)

### Sleep-state quantification

**Fig. 2:**
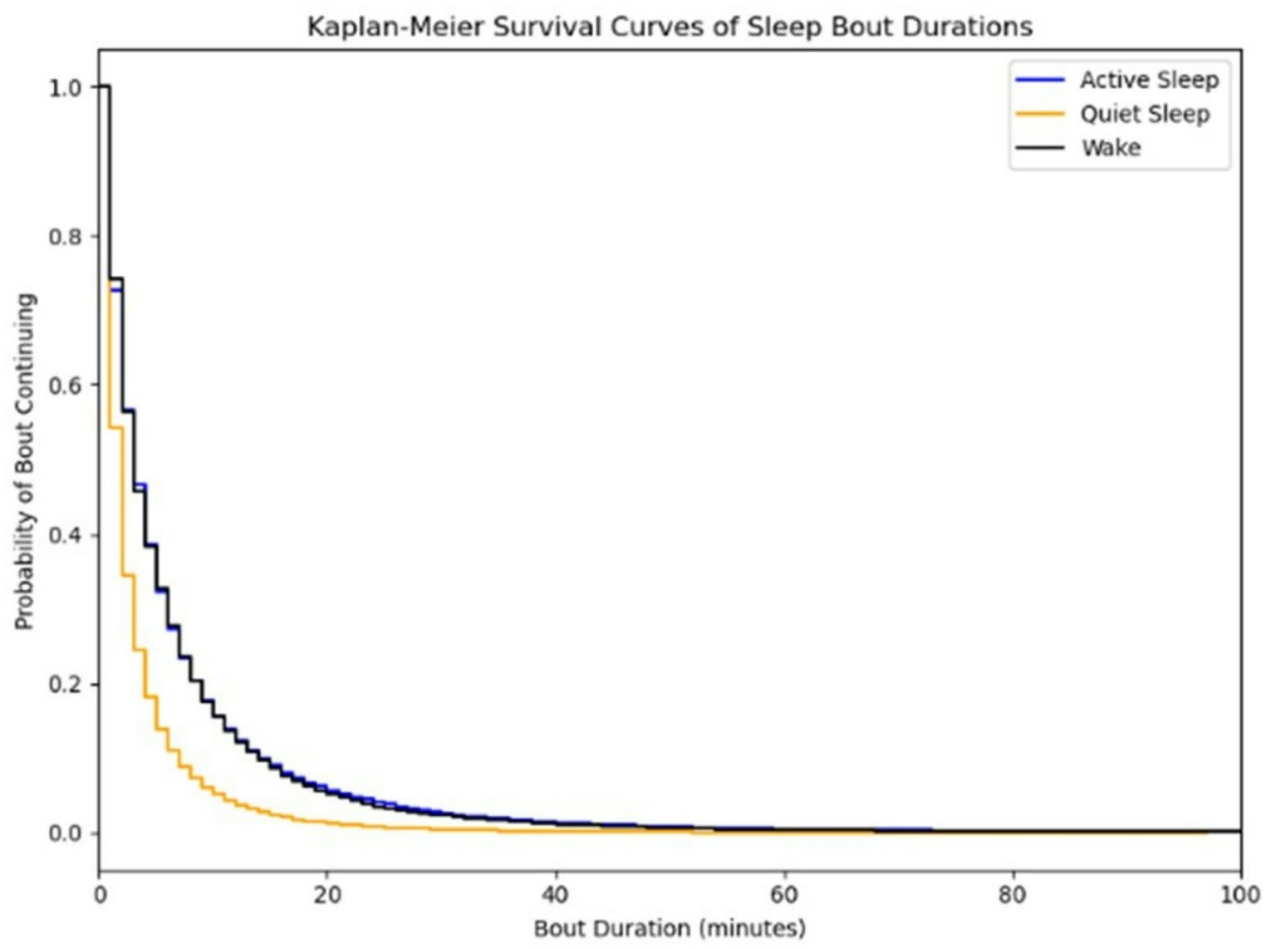
Kaplan-Meier survival curves of different sleep stages. Sleep stages are active sleep (Blue), quiet sleep (Yellow) and wake (Black). The x-axis shows bout duration in minutes (truncated at 100 min) and the y-axis shows the proportion of bouts still ongoing at each time point. Quiet sleep bouts decline faster, indicating shorter durations compared to active sleep and wake, which have similar bout durations

**Fig. 3:**
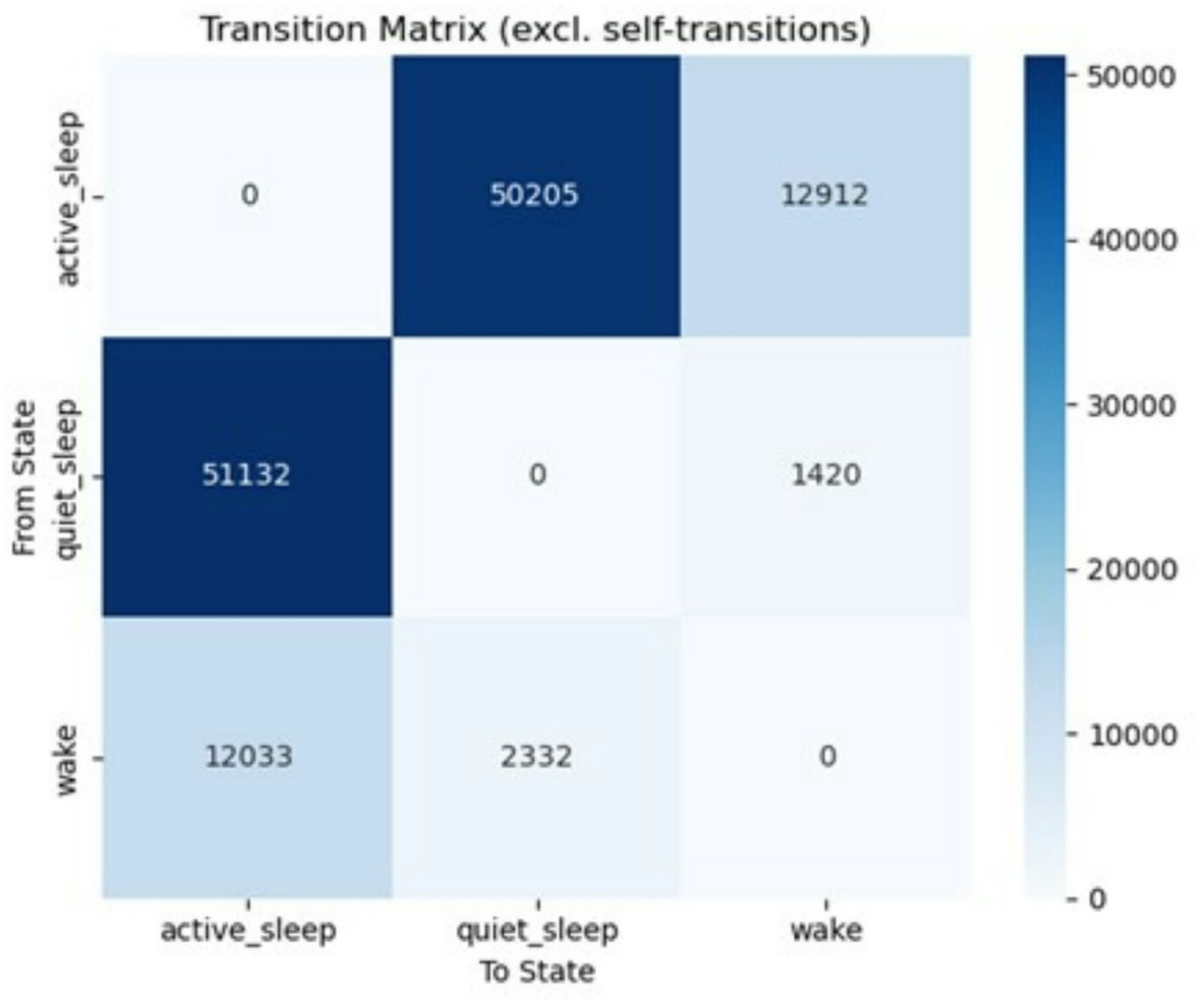
State transition matrix. Matrix showing number of transitions between behavioural states. Rows indicate the starting state, columns the resulting state. Diagonal elements (transitions within the same state) are excluded. Most transitions occurred between active and quiet sleep

We analyzed 667,069 valid minutes. As expected, sleep predominated: AS 60.0 (11.7)%, quiet sleep (QS) 25.2 (12.4)%, wake 13.4 (2.7)%; total sleep (AS + QS) 85.2 (2.7)% (Figs. 2 and 3). Mean bout durations were 6.26 (10.02) min for AS, 3.18 (4.38) min for QS, and 6.08 (8.88) min for wake. Despite frequent AS↔QS alternations (132,034 transitions), consolidated sleep blocks (uninterrupted AS/QS) averaged 38.2 (10.2) min.

### Clinical predictors of absolute cerebellar volume

**Fig. 4:**
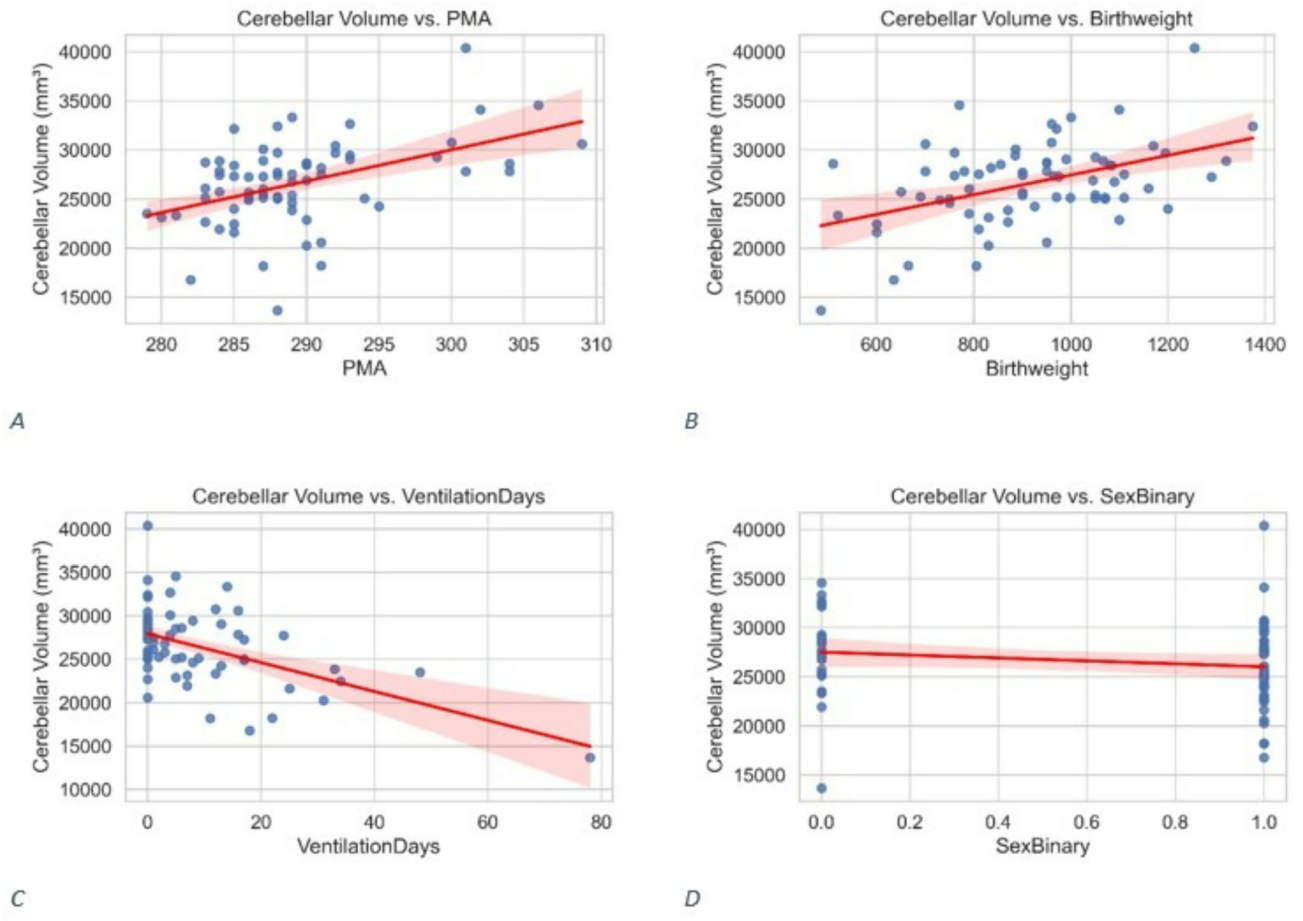
Associations between clinical predictors and cerebellar volume. Scatterplots with regression lines showing the relationship between (**A**) postmenstrual age at MRI, (**B**) birthweight, (**C**) days of ventilation and (**D**) sex (0 = male, 1 = female) with relative cerebellar volume. All variables were included in a multiple linear regression model (R² = 0.565, adjusted R² = 0.539, *p* < 0.001)

A multivariable model with absolute cerebellar volume as outcome was significant (R² = 0.565; adjusted R² = 0.539; F(4,66) = 21.47; *p* < 0.001). Larger volume was associated with higher PMA at MRI (B = 332.5, SE = 56.5; *p* < 0.001) and greater birthweight (B = 5.46, SE = 2.09; *p* = 0.011), and smaller volume with more ventilation days (B = − 116.9, SE = 30.6; *p* < 0.001) and female sex (B = − 1877.6, SE = 730.0; *p* = 0.012) (Fig. 4a–d).

### Primary association: total sleep and proportional cerebellar size

**Fig. 5:**
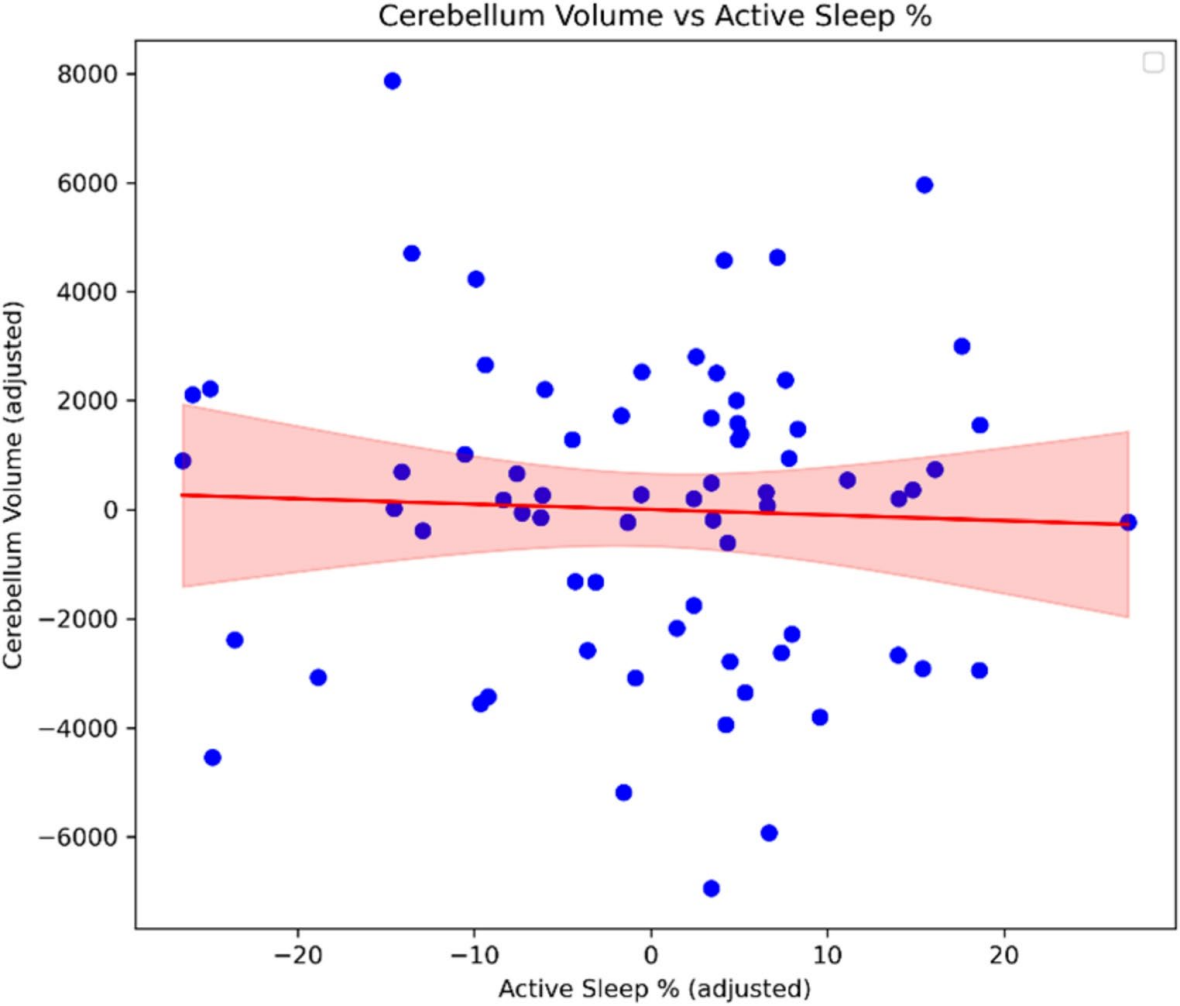
Association between total sleep percentage and cerebellar volume measures. (**A**) Scatter plot showing no significant relationship between total sleep percentage and absolute cerebellar volume (mm^3^ ) (*p* = 0.34). (**B**) Scatter plot showing a significant positive association between total sleep percentage and the cerebellum-to-total brain volume ratio (*p* = 0.011)

When cerebellar size was normalized to total brain volume (cerebellum/brain ratio), greater total sleep percentage during NICU monitoring was associated with a larger proportional cerebellar size at TEA (β = 0.54, *p* = 0.011) (Fig. 5). This effect was independent of PMA at MRI, birthweight, ventilation days, and sex.

### Secondary analyses

**Fig. 6:**
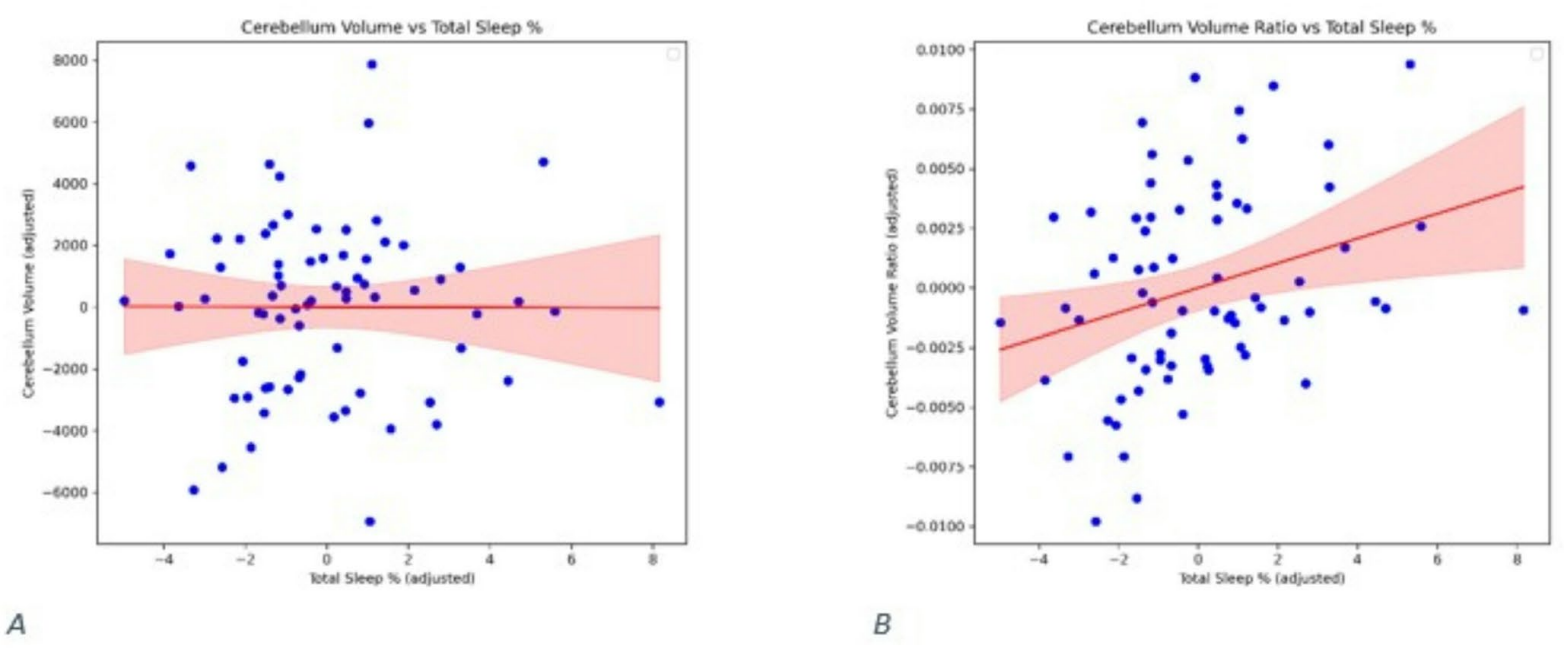
Association between active sleep percentage and cerebellar volume. Scatter plot illustrating the relationship between active sleep percentage during the second postnatal week and absolute cerebellar volume (mm^3^ ) at term-equivalent age. No significant association was observed (*p* = 0.14, β -0.31)

**Fig. 7:**
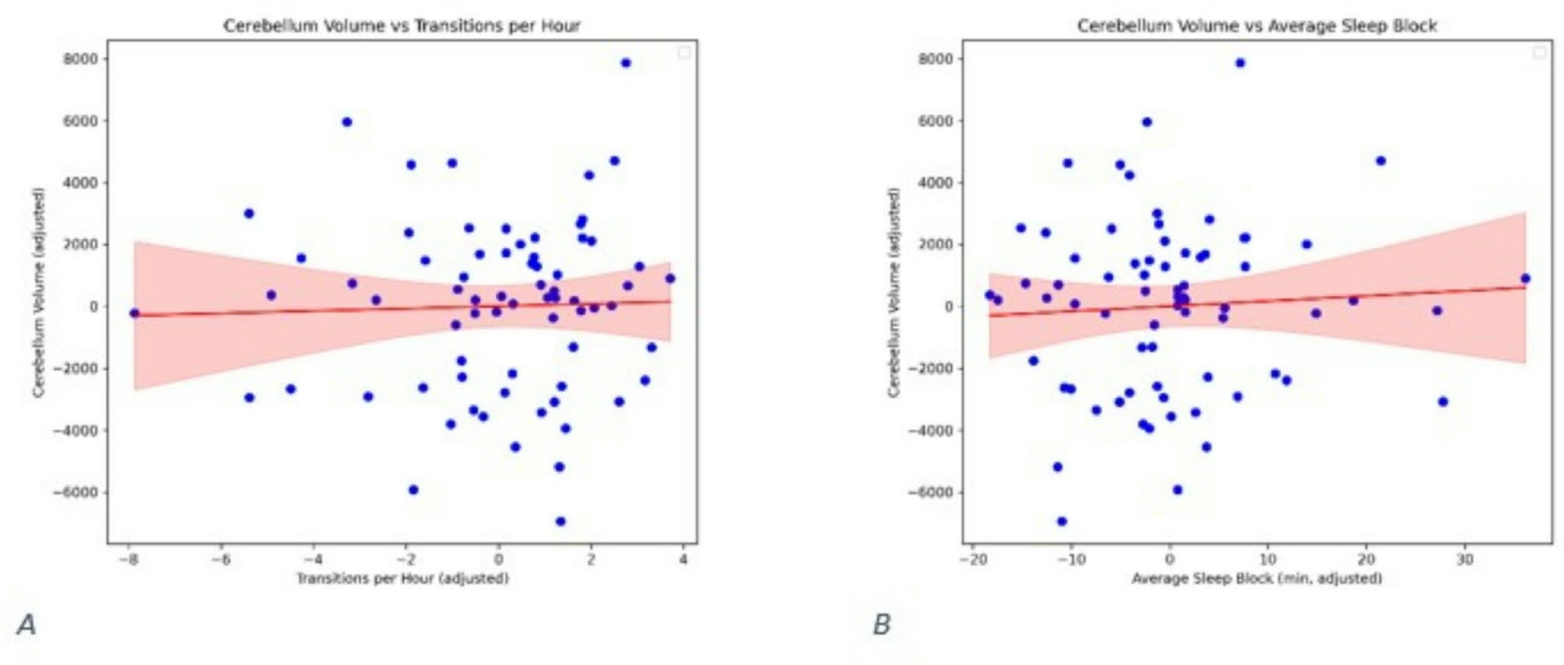
Sleep state transitions and average bout duration versus cerebellar volume. (**A**) Number of transitions between behavioural states did not show a significant association with absolute cerebellar volume (*p* = 0.847) or relative cerebellar volume (*p* = 0.792). (**B**) Average sleep bout duration was not significantly related to either absolute cerebellar volume (*p* = 0.958) or relative cerebellar volume (*p* = 0.932)

State composition alone did not relate to absolute cerebellar volume: AS% (β = −0.31, *p* = 0.14), QS% (*p* = 0.49), wake% (*p* = 0.65), or total sleep% (*p* = 0.34) (Fig. 6). Dynamic metrics (transitions per hour and mean sleep-bout duration) were not associated with absolute volume (transitions: *p* = 0.41; bout duration: *p* = 0.25) (Fig. 7).

## Discussion

In this cohort of extremely preterm infants, a higher total sleep percentage during the late second to early third trimester equivalent period (29–31 weeks PMA) was associated with a larger proportional cerebellar size at term-equivalent age, after adjustment for key clinical factors. In contrast, the proportions of active and quiet sleep considered separately were not related to absolute cerebellar volume. This pattern suggests that overall sleep quantity and continuity, rather than the relative share of individual states, may be most relevant for cerebellar growth relative to the rest of the brain in this period.

As outlined in the introduction, late gestation is characterized by rapid cerebellar expansion and by sleep as the dominant brain state providing structured spontaneous activity and a stable physiological milieu [8–10, 22, 24]. Experimental work in animals and human infants shows that active sleep–related twitches engage cerebellar and sensorimotor circuits in a way that is well suited to activity-dependent calibration, whereas quiet sleep is thought to support synaptic renormalization [14–16, 18–20, 30]. Our finding that more total sleep is linked to a relatively larger cerebellum is therefore consistent with the long-standing view of sleep as a primary driver of spontaneous brain activity with developmental significance (Roffwarg et al., 1966), rather than a passive state.

These results also emphasize that sleep should be considered explicitly in studies of brain development in preterm infants. Sleep quantity and continuity are modifiable features of NICU care and co-vary with handling, light and noise exposure, analgesia, and clinical instability [22–25]. Given the cerebellum’s involvement in both motor control and higher cognitive functions [12], sleep-aware care practices (for example protected sleep windows, clustered care, and environmental noise/light reduction) are plausible levers to support cerebellar development. Our data suggest that sleep should be modeled alongside established determinants such as nutrition, respiratory support, and inflammatory burden when interpreting cerebellar outcomes [31]. The absence of an association with absolute cerebellar volume, together with the positive relationship observed for the cerebellum-to-brain ratio, does not appear to be driven by lower total brain volume in infants with higher sleep percentages. Rather, the findings may reflect differential regional scaling between cerebellar and supratentorial growth. Given the use of global volumetric measures, this interpretation remains cautious and hypothesis-generating.

We did not find an association between the total amount of active sleep and absolute cerebellar volume at TEA, even though in the same cohort active-sleep percentage related to supratentorial white-matter measures [32, 33]. Several factors may explain this difference. First, our cerebellar endpoint was a global volume that does not separate gray and white matter at neonatal MRI resolution; any tract-specific or layer-specific effects (for example within the cerebellar peduncles or external granular layer) are likely diluted when averaged into a single total-volume measure [34, 35]. Cerebral white matter, by contrast, is larger, more readily segmented, and may be more sensitive to active-sleep–linked plasticity, allowing associations to emerge supratentorial but not within a global cerebellar metric. Second, Our sleep measurements primarily reflect the early extrauterine period around 29–31 weeks PMA; sleep organization later in the NICU course, closer to term-equivalent age, was not captured and may also contribute to cerebellar development. Third, vital-sign–based staging provides coarse state labels and cannot capture the microstructure of active sleep, such as twitch rate, clustering, and inter-twitch intervals. If these finer features are most relevant for cerebellar calibration, their influence may be obscured when using simple state proportions. Fourth, the sleep assessment was performed several weeks prior to the TEA MRI, and this temporal separation may have diluted detectable associations with absolute cerebellar growth. It is plausible that sleep measured closer to term-equivalent age would show stronger structure-sleep coupling. Longitudinal studies with repeated sleep phenotyping across the 24–40 week PMA window are needed to define potential sensitive periods.

Several limitations should be taken into account. First, our proportional outcome relied on brain–cerebellum ratios derived from whole-brain estimates that may still include non-parenchymal components; although covariate adjustment and sensitivity analyses reduce the likelihood of simple scaling effects, tissue-specific normalization would be preferable. Second, cardiorespiratory classifiers, while validated and practical at the bedside, remain surrogate measures that do not quantify cortical signatures or twitches directly, and misclassification is most likely near state transitions [28, 36]. Third, the relatively brief monitoring interval prevents assessment of cumulative sleep “dose” and possible age-dependent effects across the broader 24–40 week postmenstrual age range. Fourth, global cerebellar volume averages across lobules, layers, and peduncles; more fine-grained microstructural and tract-specific measures are likely more sensitive to subtle effects [34]. Fifth, residual confounding by unmeasured factors, such as detailed nutritional intake, standardized pain and stress exposure, and inflammatory markers, cannot be excluded [31]. Sixth, The cohort was relatively clinically stable, with no cases of necrotizing enterocolitis (NEC), no gram-negative sepsis, and no infants requiring treatment for patent ductus arteriosus (PDA). This low burden of major inflammatory and systemic morbidity reduces confounding by severe illness, but may limit generalizability to more medically complex extremely preterm populations and finally the segmentation approach provided global cerebellar volumes but did not allow reliable parcellation of vermian and hemispheric subregions. Region-specific cerebellar measures may be more sensitive to sleep-related effects and should be explored in future studies using dedicated neonatal cerebellar segmentation methods.

Future work should therefore move beyond coarse state proportions toward more mechanistic sleep phenotyping, combining lightweight EEG, high-frame-rate video for automated twitch detection, and refined cardiopulmonary metrics to derive continuity indices and twitch-based features. Imaging protocols should include region- and tract-specific endpoints, such as lobule-wise volumetry, diffusion metrics of the cerebellar peduncles, and myelin-sensitive mapping, to test whether sleep is preferentially related to anterior sensorimotor territories and cerebellar white matter [12, 34]. Longitudinal designs spanning 24–40 weeks postmenstrual age, ideally embedded in sleep-supportive intervention studies, will be needed to disentangle driver versus biomarker roles and to link intermediate imaging measures to later motor and cognitive outcomes.

## Conclusions

In summary, in extremely preterm infants more total sleep during the late second to early third trimester equivalent period (29–31 weeks PMA) is associated with a larger proportional cerebellar size at term-equivalent age, whereas active-sleep percentage alone does not predict absolute cerebellar volume. This pattern supports a model in which sleep quantity and continuity modulate cerebellar growth relative to the rest of the brain, while absolute size remains strongly influenced by systemic clinical factors. The next step is to test whether targeted, sleep-supportive NICU strategies can measurably alter cerebellar trajectories, using finely resolved sleep metrics and regional cerebellar measures as predefined outcomes. 

## Data Availability

The dataset generated and analysed during the current study are not publicly available due to patient privacy regulations. Pseudonymized data may be made available from corresponding author upon reasonable request and subject to institutional data-sharing agreements.

## Declarations

### Data privacy and confidentiality

To ensure data privacy, all collected information was pseudonymized prior to analysis and handled in accordance with institutional and national data‑protection regulations.
