# Correction to: Sleep During NICU Care Is Associated With Proportional Cerebellar Size at Term-Equivalent Age in Extremely Preterm Infants

**DOI:** 10.1007/s12311-026-02010-5

**Published:** 2026-04-30

**Authors:** J. M. Brouwer, A. M. Bakker, E. de Groot, M. L. Tataranno, M. Benders, J. Dudink

**Affiliations:** 1 Faculty of Science, University of Amsterdam, Science Park 904, Amsterdam, 1098 XH The Netherlands; 2 Department of Neonatology, Wilhelmina Children’s Hospital, University Medical Center Utrecht, Lundlaan 6, Utrecht, 3584 EA The Netherlands; 3 Faculty of Medical Sciences, University of Groningen, Antonius Deusinglaan 1, Groningen, 9713 AV The Netherlands

**Correction to: The Cerebellum (2026) 25:49**

10.1007/s12311-026-01988-2

In the original version of this article, a typo was found in the article title. "Extrem Infantsmely Preter" should be "Extremely Preterm Infants."

The original article has been corrected.

